# Comparison of Structural, Physicochemical, and Functional Properties of Blueberry Residue Dietary Fiber Extracted by Wet Ball Milling and Cross-Linking Methods

**DOI:** 10.3390/foods14071196

**Published:** 2025-03-28

**Authors:** Guihun Jiang, Kashif Ameer, Karna Ramachandraiah, Xiaoyu Feng, Xiaolu Jin, Qiaolin Tan, Xianfeng Huang

**Affiliations:** 1 School of Public Health, Jilin Medical University, Jilin 132013, China; fengxiaoyu0623@163.com (X.F.); 18679735818@163.com (X.J.); 15023557526@163.com (Q.T.); 15044272738@163.com (X.H.); 2 Institute of Food Science and Nutrition, University of Sargodha, Sargodha 40100, Pakistan; kashifameer89@gmail.com; 3 Department of Biological Sciences, College of Arts & Sciences, University of North Florida, Jacksonville, FL 32224, USA; karna.r@unf.edu

**Keywords:** *Vaccinium caesariense*, extraction methods, thermal stability, hydration and adsorption capacities

## Abstract

This study evaluated the structural, physicochemical, and functional characteristics of blueberry residue dietary fiber (DF) extracted by wet ball milling (WB) and cross-linking (C) treatments. The particle size of WB-DF showed a significant decreasing trend (*p* ≤ 0.05) compared to that of C-DF and blueberry residue. Scanning electron microscopy (SEM) demonstrated that WB treatment unfolded the flaky structure of DF and caused more pores to occur. The results showed that the modifications of WB increased the release of active groups and enhanced the hydration and adsorption capacities. X-ray diffraction (XRD) analysis showed the highest crystallinity observed for C-DF, resulting in the increased thermal stability of C-DF. The molar ratios of monosaccharides were also influenced by different modification techniques. In addition, WB-DF showed the lowest ζ-potential and highest viscosity among all samples. Conclusively, DF extracted by WB treatment exhibited remarkable application potential in the functional food industry.

## 1. Introduction

Blueberry (*Vaccinium caesariense*) is a perennial plant that belongs to the *Ericaceae* family classified under *Vaccinium* genus and produces blue or purple berries. The blueberry is widely cultivated in Asia, North America, and Europe [1]. As far as nutritional composition is concerned, 100 g of blueberry comprises carbohydrates (14.5 g), protein (0.7 g), fat (0.3 g), and water (84%). One cup (148 g) serving of blueberries provides about 25% of the recommended daily intake of vitamin C and 3.6 g of dietary fiber with only 80 calories [2,3]. Blueberries are a good source of vitamins and minerals, such as vitamin K, vitamin C, and manganese [4]. Moreover, blueberries are rich in antioxidant compounds including anthocyanins, quercetin, and myricetin [2,5]. Regular consumption of blueberries provides several health benefits, such as the prevention of heart disease, brain health improvement, and the regulation of blood sugar levels [1,6]. Not only are blueberries consumed in their raw form, but they are also widely processed into a variety of products like jam, fruit vinegar, fruit wine, and fruit juice. The processing of blueberries results in the production of a significant number of by-products including fruit peels and pomace, which leads to the waste of resources and the degradation of the environment. According to research conducted by Lei et al. [7], blueberry residue has a significant amount of dietary fiber (DF), with a fiber level of 13.32% and a hemicellulose content of 6.53%. In order to significantly boost the utilization rate of blueberries, it is possible to successfully extract DF from them.

DF is classified as a carbohydrate polymer that does not undergo any hydrolysis by endogenous digestive enzymes in the small intestine; however, gut microbiota in the large intestine play a pivotal role by partial or complete DF fermentation [8]. DF exhibits several health benefits as a major bioactive compound or functional ingredient in food processing. Depending on solubility, DF is characterized as soluble dietary fiber (SDF) and insoluble dietary fiber (IDF). IDF usually includes hemicellulose, cellulose, and lignin; whereas, mucilage, gums, and pectic substances are included in SDF [9,10]. SDF, as a functional food ingredient, is employed in the food industry owing to its superior water-holding capacity and solubility. SDF is also used as a thickener, fat substitute, stabilizer, and emulsifier to improve the mouthfeel and storage stability of food products [11]. On the other hand, IDF has been shown to promote intestinal peristalsis and fecal volume, decrease the transit time in the intestines, and prevent obesity [10,12,13].

Current extraction methods including physical, chemical, and biological techniques to obtain SDF may lead to partial breakage of insoluble glycosidic bonds to some extent and, hence, cause improvement in the SDF extraction rate [14]. Different extraction methods not only result in change in DF yield but may also lead to significant changes in composition and structural characteristics of DF and, hence, the modification in its physicochemical and functional properties [15,16,17,18]. Among these methods, cross-linking (C) is a chemical DF extraction technique that involves the inclusion of phosphate groups in DF chains, which results in the enhanced development of network structure, hence, leading to improved physicochemical properties of DF [16].

Ball milling is a physical technique to modify DF properties and is one effective way to obtain micro- and ultrafine powders with excellent functional properties, such as increasing tendencies in solubility and antioxidant property [19]. Ball milling may also cause significant changes in physicochemical properties of raw materials through synergistic action of impact force, friction, and collision. Ball milling procedures include wet and dry treatments. Compared to that of dry ball milling, it is possible to obtain powder particles through wet ball milling (WB), having uniform particle size distribution [20]. In the WB process, the polysaccharides interact with water molecules and, hence, along with collision with container walls and balls, the heat is generated as a result of friction. This friction heat plays a vital role in the alternation of polysaccharides’ properties [21].

Currently, supportive evidence is not available in the published literature on both C and WB treatments when compared with each other regarding the effects on physicochemical and functional properties of blueberry residue DF. This study aimed to investigate the effects of C and WB extraction techniques on physicochemical, structural, and functional properties of blueberry residue DF. Moreover, the DF fractions extracted by C and WB methods were compared to that of untreated blueberry residue. This work may be helpful to obtain information regarding DF research on blueberry residue, hence, creating a theoretical foundation for the thorough processing and modification of blueberry residue DF for useful food additive manufacturing. Moreover, the blueberry residue DF might be utilized in the food industry with appreciable functional and physiological attributes.

## 2. Materials and Methods

### 2.1. Materials

Procurement of fresh blueberry fruits was carried out from the local supermarket in Jilin, China. To ensure consistency, only fruits with uniform ripeness (determined by firmness and color), similar size (1.5 cm in length), and absence of physical defects were selected. The moisture content and soluble solids content of the blueberries were 86% and 10.52°Brix. Acetonitrile and monosaccharide components were purchased from Sigma-Aldrich (St. Louis, MO, USA). Sodium tri-metaphosphate, sodium tri-polyphosphate, triflouroacetic acid, acetonitrile, sodium hydrogenphosphate, and potassium phosphate monobasic were purchased from Shanghai Yuanye Biotechnology Co., Ltd., Shanghai, China. The reagents and chemicals employed in this study were of analytical grade.

### 2.2. Extraction of Blueberry Residue DF

#### Preparation of Blueberry Residue

Initially, the blueberry fruits were washed thoroughly and then subjected to chopping. A JYL-C020E blender (Joyoung Co. Ltd., Jinan, China) was used to grind the chopped fruits for 1 min. Blueberry residue was obtained by filtering the juice. Using an oven drier (202-1, Taisite Instrument Co., Ltd., Tianjin, China), the residue was evenly spread out on square plastic plates (12 cm × 12 cm × 2 cm) and dried at 60 °C until the final moisture content was lowered to 5–7%. The moisture content (MC) on a wet basis was calculated based on the following formula:

(1) $$MC(\%)=\frac{Wi-Wf}{Wi}\times 100$$

where *Wi* = Initial weight of the sample, *Wf* = Final dry weight of the sample.

Finally, grinding of the dried samples was carried out by passing through the 60-mesh sieves to complete sifting to obtain blueberry residue powder. Then, the finely ground samples of blueberry residue powder were packed in laminated bags and sealed.

### 2.3. WB Extraction of Blueberry Residue DF

The blueberry residue powder was taken in a specified amount of 2 g and then, the reaction mixture was prepared by mixing with 100 mL distilled water and resultant water–fiber suspension was obtained. Then, a planetary ball mill (YXQM-1 L, Mitr Inst. & Equip. Co., Ltd., Changsha, China) was employed. The planetary ball mill contained agate milling jar (500 mL). The suspension was added with two big agate balls with a diameter of 1.5 cm, eight medium agate balls (0.8 cm diameter), and ten small agate balls with a diameter of 0.5 cm. The mill was processed in a horizontal manner at a rotational speed of 300 rpm for a time interval of 10 min. After every 5 min, the rotational direction of the ball mill was changed. After the completion of milling treatment, the balls were removed. Then, collected samples were mixed with ethanol (1:4, *v*/*v*) and allowed to stand for 4 h. After that, the residue was subjected to oven drying at 50 °C for 5 h after completion of the standing period of 12 h. Then, dried powders were sealed in laminated air-tight bags and stored until further analysis. The DF extracted by WB was termed as WB-DF. Equation (1), mentioned below, was used to calculate the blueberry residue DF yield.

(2) $$Yield\;(\%)=\frac{D}{W}\times 100\;$$

In Equation (2), *D* is indicative of the weight of DF, whereby *W* is indicative of blueberry residue powder.

### 2.4. C Extraction of Blueberry Residue DF

The method reported by Kanwar et al. [16] was employed to carry out C treatment for blueberry residue DF. For blueberry residue DF extraction by C, different C agents, such as sodium tri-metaphosphate/sodium tri-polyphosphate, were employed in a ratio of 99:1%. The DF extracted by C was termed as C-DF. The extraction yield (%) was determined as per Equation (1). Equation (2) (provided below) was used to measure the degree of C.

(3) $$Cross-linking\;degree=\frac{A-B}{A}\times 100$$

whereby *A* denoted peak viscosity of native DF and *B* was indicative of the peak viscosity of cross-linked DF.

### 2.5. Proximate Composition

All samples were analyzed for their proximate composition parameters, such as protein (AOAC Method 945.18-B), ash (AOAC Method 942.05), and fat (AOAC Method 948.22) according to the AOAC methods [22].

### 2.6. Scanning Electron Microscopy (SEM)

WB and C methods affected the microstructural and morphological attributes of DF fractions, and these effects were examined by the SEM (SU8010, Hitachi, Tokyo, Japan). All samples were attached to the specimen holder. Then, each sample was subjected to plating on gold sputter using gold powder. Then, samples were analyzed by SEM analysis at an operational voltage of 5 kV, whereby SEM imagery recording was carried out at 1000× magnification level.

### 2.7. FTIR Spectroscopy

Blueberry residue DF samples comprise different functional groups in their composition and these functional groups were analyzed in DF samples by FTIR spectroscopy. To perform FTIR analysis, the employed spectrum wavelength range was 400–4000 cm^−1^ for the FTIR spectrophotometer (Tensor 27, Bruker Daltonics Inc., Bremen, Germany) and the scan speed and resolution were <10 s and 4 cm^−1^ for 32 scans, respectively. Finally, the FTIR spectra were recorded by carrying out spectroscopy in Attenuated Total Reflection (ATR) mode.

### 2.8. Thermal Stability

All samples (5 mg each) were evaluated for their thermal properties in terms of weight loss. The weight loss measurement was carried out using a thermogravimetric analyzer (TGA, TG/DTA 8122, Rigaku, Tokyo, Japan) according to the reported method of Jiang et al. [15]. The TGA analysis was performed at an operational temperature range of 30–600 °C under the inert nitrogen environment at the heating rate of 20 °C/min.

### 2.9. X-Ray Diffraction (XRD) Analysis

All samples were subjected to XRD analysis using the X-ray diffractometer (7000S, Shimadzu, Kyoto, Japan). For XRD analysis, 40 kV copper Kα radiation (50 mA and 0.154 nm) was used, whereas the employed diffraction angle (2θ) ranged from 4 to 40°. Relative crystallinity was calculated using Origin 2019 software.

### 2.10. Rheological Measurements

The blueberry residue DF samples were analyzed for viscosity measurements by rheometer (Discovery HR-1, TA, New Castle, DE, USA) at an ambient temperature of 25 °C. The shear rate of the rheometer was varied in range from 0 to 100 s^−1^. Different rheological parameters, such as apparent viscosity (γ̇), consistent coefficient (K), and flow behavior index (n) were also assessed in accordance with the Power Law model for BR, C-DF, and WB-DF fractions by following the method of Jiang et al. [23].

### 2.11. Particle Size and Zeta Potential Determination

The particle sizes of blueberry residue DF samples were determined, along with the polydispersity index by means of Zeta Sizer (Nano ZS, Malvern Instr., Malvern, UK). For measurement purposes, the average data were recorded for a total of six measurements.

Blueberry residue DF samples were also assessed for their respective zeta potential by Zeta Sizer and the basis for estimation of zeta potential was electrophoretic mobility under an applied electric field. Moreover, the average data were recorded for a total of 30 measurements.

### 2.12. Monosaccharides Determination

The reported method of Jiang et al. [24] was used for the determination of monosaccharides through the HPLC technique (Dionex Thermo Ultimate 3000, Dionex Co., Sunnyvale, CA, USA). For analysis, each sample was accurately weighed by taking an amount of 2 mg, and then subjected to dissolution in 1 mL of triflouroacetic acid (TFA: 2 M). This mixing process took 2 h and was carried out in a hydrothermal reactor at 120 °C. Before carrying out HPLC analysis, a 0.45 µm membrane filter was used to filter the aqueous layer from all DF samples in conjunction with a DAD detector (Thermo Fisher Sci, Waltham, MA, USA). Mobile phase employed for analysis was prepared by the dissolution of acetonitrile (A) and phosphate buffer (PBS: 0.1 mol/mL and pH 6.7) solutions in a mixing ratio of 82:18 (*v*/*v*). ODS2 Supersil column (4.6 × 250 mm^2^, 5 μm) was used for analytical procedure while the injection volume and flow rate were 20 µL and 0.8 mL/min, respectively. The detection of monosaccharides was performed against reference standards at specified detection wavelength of 245 nm.

### 2.13. Hydration Properties

The water holding capacity (WHC), oil holding capacity (OHC), and water swelling capacity (WSC) were determined as per the procedures published by Raza et al. [25] and He et al. [26], respectively.

### 2.14. Functional Properties

The bile acid-adsorption capacity (BAC), cholesterol adsorption capacity (CAC), and glucose adsorption capacity (GAC) were assessed according to the procedures of Luo et al. [27] and Wang et al. [28], respectively.

### 2.15. Statistical Analysis

All measurements and each extraction treatment were recorded in triplicate (n = 3), and three replications were performed. The data were subjected to one-way analysis of variance (ANOVA) to determine differences between treatments using SPSS 20.0 (Chicago, IL, USA). A two-way ANOVA was also performed for statistical comparisons of modification methods and pH, as well as modification methods and glucose concentration. Mean comparison was carried out using Duncan’s multiple-range test at *p* ≤ 0.05.

## 3. Results and Discussion

### 3.1. DF Extraction Yield and Proximate Composition

The results of extraction yield and proximate composition of blueberry residue DF samples are provided in Table 1. The degree of C of modified DF was 2.34%, demonstrating that a portion of free hydroxyl groups of DFs was successfully replaced by the modifying phosphate group. For the extraction yield, the C-DF fraction (71.78%) was higher than that of WB-DF fraction (40.62%). The higher DF yield in the case of C-DF could possibly be ascribed to cell wall shear breakdown in conjunction with disruption of the densely packed molecules, as well as the structural configuration induced by C agents. Moreover, this C treatment also caused dissolution of IDF hemicellulose in a partial manner, which consequently led to achieving the highest DF yield as compared to those of WB-DF. The C treatment may result in the formation of cross-links with fiber molecules, which might reduce the loss of fiber during processing and, hence, may lead to higher DF yield [16]. In the case of protein, the blueberry residue had the highest protein content of 0.94% when compared to those of WB-DF (0.54%) and C-DF (0.45%) fractions of blueberry residue. Among all samples, the blueberry residue had the highest ash and fat contents, followed by WB-DF and C-DF fractions. The slightly reducing tendencies in protein, fat, and ash content of C-DF and WB-DF fractions in comparison with those of blueberry residue could be due to the probable varying degree of fiber matrix degradation during the modification process [29].

### 3.2. SEM

**Figure 1:**
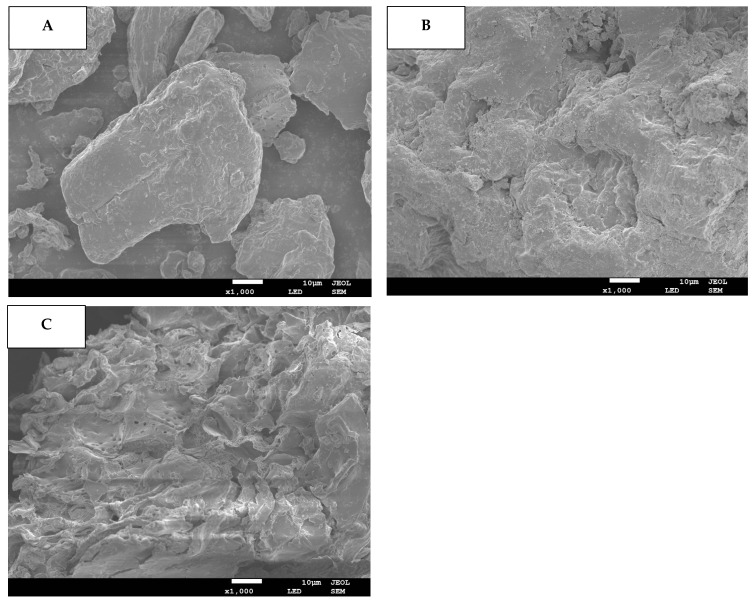
SEM images for BR (**A**), C-DF (**B**), and WB-DF (**C**). BR: blueberry residue; C-DF: cross-linked dietary fiber of blueberry residue; WB-DF: wet ball-milled dietary fiber of blueberry residue.

The DF samples’ microstructural attributes were assessed through SEM and results are provided for blueberry residue (Figure 1A) as well as blueberry residue DF fractions extracted by C (C-DF: Figure 1B) and WB (WB-DF: Figure 1C). When compared with WB-DF and C-DF, the blueberry residue had a dense texture and smooth surface with no existence of pores. However, the C-DF (Figure 1B) and WB-DF (Figure 1C) had surface irregularity in conjunction with microstructural damage, enhanced pore loosening, and degradation of cell walls. In the case of plant cell walls, the microfibrils play a vital role in making up the structural framework constituents of cell walls, such as lignin, cellulose, and hemicellulose [30,31]. Both lignin and hemicellulose play contributory roles as binder and filler between these microfibrils. The cracks and fissures were also visible on the surfaces of treated C-DF and WB-DF blueberry residue DF fractions. WB-DF fraction exhibited an increased presence of pore loosening, surface irregularities, fissures and cracks, microstructure impairment, and cell wall disintegration compared to DF fraction obtained by C-DF. In contrast to C-DF, the rising tendency of porosity in the case of the WB-DF structure might be attributable to the shearing and collisions owing to WB treatment, which was in agreement with the previously reported finding of Ma and Mu [31]. Figure 1C also depicted the WB treatment, which caused significant enlargement in surface area of DF as compared to that of C treatment, hence, resulting in enhanced binding and water absorption. Conclusively, WB-DF exhibited the maximum alteration in the apparent structure of DF as compared to that of C-DF and blueberry residue. WB treatment increased the degree of formation of cavities in WB-DF, thereby resulting in enhanced specific area and improved physicochemical and functional characteristics.

### 3.3. FTIR

**Figure 2:**
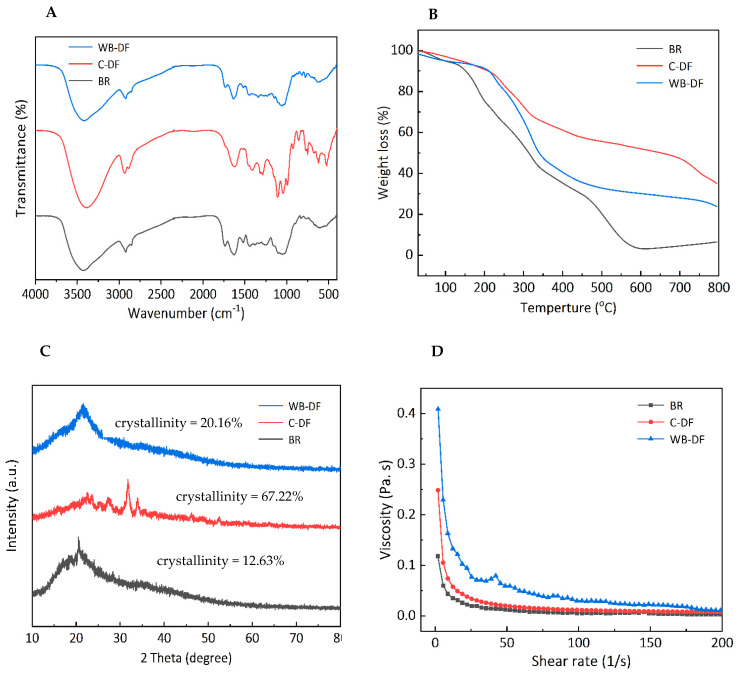
FT-IR spectra (**A**), thermal properties (**B**), X-ray diffraction (**C**), and rheogram plot (**D**) for BR, C-DF, and WB-DF. BR: blueberry residue; C-DF: cross-linked dietary fiber of blueberry residue; WB-DF: wet ball-milled dietary fiber of blueberry residue.

FTIR spectroscopy was performed for all samples and characteristic peaks for blueberry residue, C-DF and WB-DF are demonstrated in Figure 2A. It was evident from results that, irrespective of the modification method employed, all DF samples exhibited two significant peaks at characteristic IR regions of 3471 cm^−1^ and 2952 cm^−1^. The absorption peaks at IR regions of 3471 cm^−1^ and 2952 cm^−1^ were indicative of the sugar methyl (–CH), hydroxyl (–OH), and methylene groups, respectively [32]. The IR vibrational peaks in IR spectral regions ranging from 3414 to 3480 cm^−1^ possibly indicated the –OH groups, mainly originating from the cellulose and hemicellulose. The peak intensity and absorption band was relatively reduced in WB-DF, which could be attributed to the destruction of molecular bonds, particularly hydrogen bond breakage in structural configurations of cellulose crystalline domain and hemicellulose chains. All samples exhibited a slight ridge of FTIR peak with a faint intensity at IR regions ranging from 2856 to 2923 cm^−1^, and these peaks corresponded to the C–H stretching and IR vibrations of –CH and –CH_2_ polysaccharides groups [33]. The absorption intensity of WB-DF samples was relatively lower at 2927 cm^−1^, which might be because of polysaccharides content reduction and hydrogen linkages disruption existing between –OH groups in the crystalline structure of cellulose. The stretching of hemicellulose, lignin, and pectin’s ester carbonyl groups was most probably responsible for the disappearance of the C-DF absorption peak at 1730 cm^−1^ [34]. It disappeared as a result of the ester linkages being broken by the sodium tri-metaphosphate/sodium tri-polyphosphate solution in an alkaline environment. Irrespective of the modification method employed, all DF samples showed IR absorption peak at an IR spectral region of 1739 cm^−1^, which indicated the possible presence of uronic ester groups originated from hemicelluloses. All DF samples showed IR peak at a spectral region of 1690 cm^−1^, which corresponded to the stretching or bending vibration of aromatic lignin hydrocarbon (aromatic benzene). The intensity of this IR spectral region exhibited decreasing tendency in WB-DF samples, which might be ascribed to the splitting of hydrogen linkages in polysaccharides configuration and the disruption of lignin molecules owing to WB treatment. The enhanced peak intensity at 1445 cm^−1^ for C-DF could be owed to the damage imparted on the crystalline area of cellulose after chemical treatment [35]. In comparison to the untreated SDF, Si et al. [36] similarly observed an increase in peak intensity in the modified tea SDF following modification. The spectral peak (IR vibration) arose at an IR spectral vibration of 1028 cm^−1^ corresponding to C–O linkages in the hemicellulose and lignin pyranose ring [37]. This IR peak was significantly higher in C-DF compared to that of WB-DF, indicating that WB treatment caused enhanced degradation of cellulose and hemicellulose in DF structure. The emergence of the peak at 1237 cm^−1^ suggested the presence of P=O bonds in cross-linked DF. Irrespective of the modification method used, all samples exhibited absorption peaks at IR spectral regions ranging from 700 to 1100 cm^−1^, and this corresponded to the presence of IR contraction vibration pertaining to C–O esters. The DF samples showed an IR stretching vibration peak of xylan at an IR region of 1000 cm^−1^, which corresponded to the C–O stretching band existing in structural linkages of C–O–C, which confirmed that DF composition comprises xylan hemicellulose.

In summary, the structure of C-DF and WB-DF was slightly different. The WB and C treatment may cause different hydration properties and adsorption capacity, resulting in different intensities of some absorption peaks.

### 3.4. Thermal Properties

All DF samples were analyzed by means of thermogravimetric analysis and results are presented in Figure 2B in terms of weight loss percentage. TGA analysis usually involves three stages. The initial stage was carried out at a thermal constraint of 30–210 °C. During 30–210 °C, the decreasing tendency was observed in all samples. Blueberry residue had the highest weight loss when compared to WB-DF and C-DF blueberry residue DF fractions, which coincided with devolatilization occurrence at 120 °C. The weight loss during the initial stage could be ascribed to the evaporation of absorbed water from DFs of blueberry residue. The second stage of TGA analysis ranged from 210 to 400 °C and the maximum weight loss occurred at this stage. The highest weight loss during the second stage was observed in the case of blueberry residue followed by WB-DF, while weight loss was lowest in C-DF in comparison with WB-DF. This might be attributable to the augmented degradation of pyrolytic polysaccharides, mainly comprising hemicellulose and pectic polysaccharides [38]. According to Qi et al. [39], thermal stability is caused by bonds found in the crystalline region of cellulose chains. The reactive groups of DFs changed following modification, as shown by the FTIR spectra (Figure 2A). According to Zhang et al. [40], the breakdown of some lignin and complex polymer components was the cause of the comparatively slow decline between 400 and 600 °C. The last stage of TGA varied from 600 to 800 °C mainly due to the loss of some macromolecule material. At this stage, the samples exhibited decreases in weight loss in a gradual manner. It was implied from the results that C-DF had the highest thermal stability followed by WB-DF and blueberry residue. Because the WB process created intermolecular crosslinks that may have increased the surface charge and colloidal dispersibility of okara and changed crystalline polysaccharides into amorphous ones, the WB-DF exhibited greater thermal stability than blueberry residue [38]. Moreover, the C-DF had a maximum thermal among all samples, which can be associated with its high crystallinity (Figure 2C).

### 3.5. XRD

The diffractograms for blueberry residue, C-DF, and WB-DF are demonstrated in Figure 2C. Generally, all samples showed main signal peaks at 2θ angles of 21.5°, 22.32°, 23.5–24.5°, and 30.8–31.9°. WB-DF showed a main signal peak in the range of 21.5–23.8°. C-DF exhibited main signal peaks at 2θ angles of 28.3°, 34.6°, and 36.7°. The degree of amorphousness was relatively high in WB-DF samples when compared with C-DF. The published literature has also reported that signal peaks exist in ranges of 14.8–15.2° and 21.5–23.8°, which could be ascribed to cellulose crystals existence. When compared with that of C-DF, WB-DF did not show front diffraction peaks at 2θ angles of 28.3°, 34.6°, and 36.7°. The front diffraction angles at 2θ angles of 21.2–23.6° in WB-DF showed a moderate degree of sharpness compared to that of C-DF. Irrespective of the modification methods, the peak breadth and positions of XRD diffractograms were significantly influenced by C and WB methods, which implied that these techniques resulted in major disruptions in DFs crystalline structures.

The crystallinity percentage for blueberry residue, C-DF, and WB-DF was 12.63%, 67.23%, and 20.16%, respectively. Enhanced crystallinity boosts the thermal stability of DF, hence, facilitating its application in food products subjected to high temperatures. When compared to blueberry residue, the crystallinity of DFs enhanced during WB, likely due to the breakdown of some amorphous components, including amorphous cellulose and hemicellulose. The increased crystallinity in C-DF may be attributed to the disturbance of the amorphous region during cross-linking and the removal of a fraction of hemicellulose and lignin [16]. Prior research indicated that the elimination of hemicellulose, lignin, or the amorphous region in cellulose could exacerbate structural damage to IDF, resulting in increased crystallinity [41]. Consequently, the rise in crystallinity of C-DF and WB-DF signifies more pronounced structural damage in DFs.

### 3.6. Viscosity

All DF samples were subjected to viscosity measurements and results are demonstrated in Figure 2D for blueberry residue, C-DF, and WB-DF in terms of rheograms. Blueberry residue and C-DF exhibited similar rheograms with few differences. All samples exhibited a viscosity decreasing trend with a corresponding rise in the applied shear rate. Both blueberry residue and C-DF had similar curvature regarding decreasing trends in their viscosities. The viscosities of blueberry residue and C-DF samples were found in ranges of 0.05–0.23 Pa.s and 0.12–0.36 Pa.s, respectively. WB-DF samples had a maximum viscosity ranging from 0.19 to 0.43 Pa.s, and it could be implied that WB treatment led to the improvement in blueberry residue DF viscosity to some extent. WB-DF had the highest value of apparent viscosity (77.08 m.Pa.s) followed by C-DF (33.81 m.Pa s) and blueberry residue (19.87 m.Pa.s) fractions (Table 2). Compared to blueberry residue, C-DF had a higher viscosity, suggesting that C treatment could somewhat increase the viscosity of the DF dispersion solution. After being treated with WB, the viscosity of modified DF was maximum. This reason could be due to WB effect, which tended to increase exposure of polar groups and enhanced WB-DF surface charge. This also caused rising tendencies in electrostatic interactions between solvent and molecules and, hence, increased the flow resistance of the solution [24]. Furthermore, the viscosity of WB-DF likely increased due to its higher negative charge density compared to other DF samples (Table 3). On the other hand, WB-DF had the highest consistency coefficient of 697.35 m.Pa.s, followed by C-DF (387.94 m.Pa.s). The flow behavior index values of blueberry residue and WB-DF samples were comparable to each other, while C-DF had a slightly lower value of flow behavior index (0.27). Conclusively, the variability in viscosities of blueberry residue DFs could be ascribed to enhanced particle interactions, cluster structure changes in blueberry residue DFs, partial branching degradation, rising tendencies in apparent viscosity, and structural modifications [42].

### 3.7. Particle Size and Zeta Potential

The results pertaining to average particle sizes of blueberry residue, WB-DF, and C-DF are presented in Table 3. Particle size of all samples was found to be in the range of 451–570 nm. The WB-DF exhibited significantly (*p* ≤ 0.05) declining tendency in particle size as compared to blueberry residue and C-DF. The significant decrease in WB-DF particle sizes compared to other fractions might be ascribed to possible disaggregation and random dissociation of blueberry residue DF structure and shearing action owing to WB treatment. WB treatment also led to the dispersion of blueberry residue DF extracts and consequently smaller particle sizes [43]. These results are in line with the findings reported by Jiang et al. [15], whereby authors reported that particle sizes of ginseng residue decreased after exposure to enzymatic WB treatment.

The zeta potential usually indicates the presence of a charge on surface materials, which is linked with DF gelation capacity. Moreover, high surface-charge presence also represents a tendency to merge with cations, and a high dissociation degree is linked with high electrostatic charge. The results of ζ-potential for all samples are provided in Table 3. ζ-potential of all samples ranged from −20.1 to −34.0 mV. In this research, WB-DF had the ζ-potential value of −34.0 mV, which was the most negative charge compared to blueberry residue (−20.1 mV) and C-DF (−28.7 mV). The published literature has already reported on the increased presence of a negative charge and its link with high electrostatic force and molecular chain extension. This leads to an increasing tendency in intermolecular C, which consequently results in stronger gel formation. Moreover, surface charge also significantly affects the blueberry residue DF stability in aqueous solutions as well as dispersion capability [44]. The results of the current study were in coherence with the previously reported findings by Bhatt and Gupta [44] and Jiang et al. [24] regarding DF extracted from mango, pomegranate peel, and papaya (*Carica papaya* L).

### 3.8. Molar Ratio of Monosaccharide Components

All samples were subjected to HPLC analysis for determination of monosaccharide composition and results are given in Table 4. It was evident from the results that Glc and GalA were the most abundant monosaccharides constituents in extracted blueberry residue DF composition. The primary sources of glucose include starch and cellulose. Among all samples, WB-DF exhibited the lowest molar ratio of Glc (5.87) as compared to C-DF (10.05) and blueberry residue (40.0) fractions. The lowest Glc molar ratio in WB-DF could be attributed to the shearing action of WB treatment, which possibly degraded the partial cellulose. Moreover, GalA has been reported to exist as one of main structural components of pectin. Blueberry residue had the lowest molar ratio of GalA (5.80), whereas C-DF had the highest GalA molar ratio (17.99) followed by WB-DF (14.90). Pectin comprises GalA in its composition as one of main components. The decreasing tendency in the molar ratio of GalA of WB-DF might be ascribed to the possible degradation of pectin and decreases in pectin content [45]. C-DF exhibited higher GalA molar ratio, which indicated that C treatment rendered DFs with optimal functional and physical properties. C-DF and WB-DF had the highest Gal and Xyl molar ratios compared to those of the blueberry residue. Higher Xyl ratio in C-DF and WB-DF possibly suggested that DFs extracted by C and WB had higher xylan hemicellulose. Overall, WB-DF fraction exhibited lower molar ratios of Rha, GalA, and Glc, which could be attributed to the likely breakdown of the glycosidic linkages [38]. Moreover, the findings of this study imply that significant alterations in monosaccharides molar ratios were observed, depending on the type of modification method.

### 3.9. Hydration and Functional Properties

#### 3.9.1. WHC, OHC, and WSC

**Figure 3:**
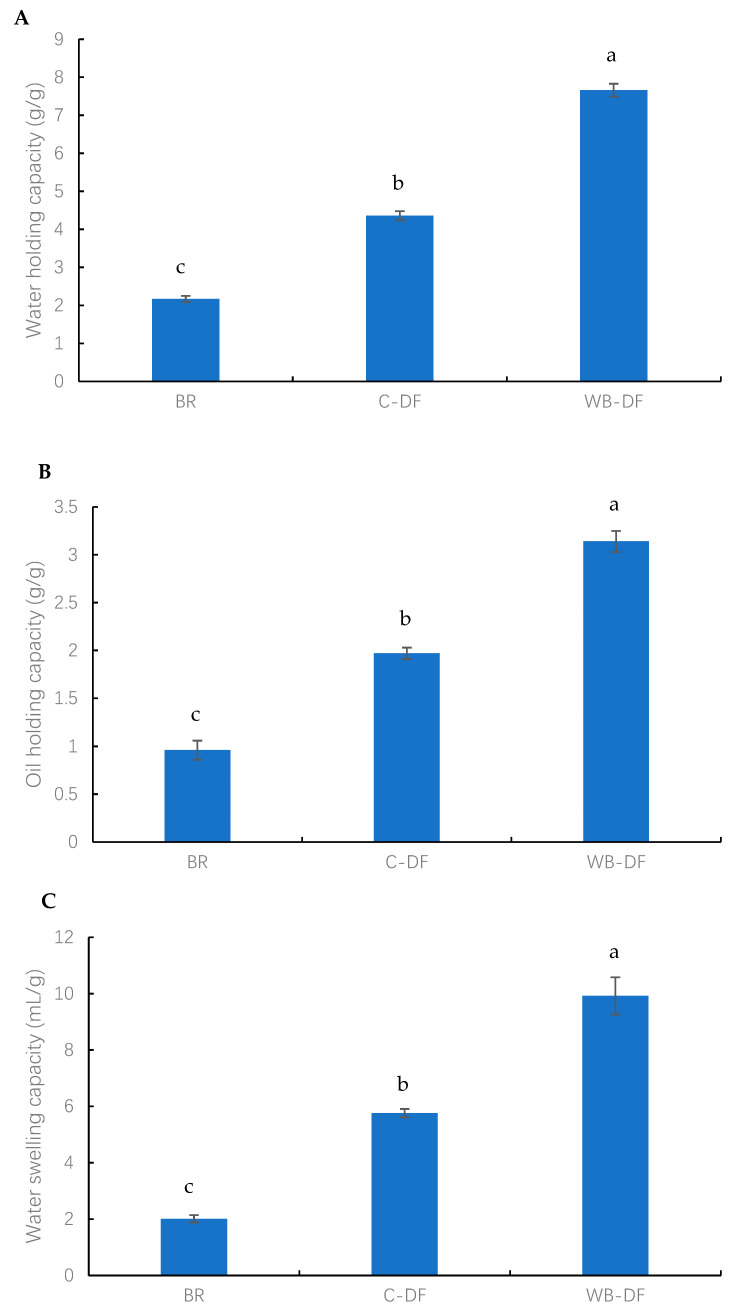
(**A**) water holding capacity (**B**) oil holding capacity (**C**) water swelling capacity of BR, C-DF, and WB-DF; BR: blueberry residue; C-DF: cross-linked dietary fiber of blueberry; WB-DF: wet ball-milled dietary fiber of blueberry residue. Different letters above the bars indicate significant differences between the mean values (*p* ≤ 0.05).

The results of WHC, OHC, and WSC of all samples (blueberry residue, C-DF, and WB-DF) are presented in Figure 3. The highest WHC was exhibited by WB-DF samples (7.67 g/g), followed by C-DF (4.36 g/g), and blueberry residue (2.17 g/g) fractions (Figure 3A). By dissolving the fiber network and creating the complex structure, Yan et al. [46] showed how chemical treatment can alter the hydration properties of DF. Moreover, WB treatment effectively led to a rise in WHC owing to decreases in particle size and the disruption of the fiber structure. Mechanical milling under aqueous conditions led to the fiber matrix breakdown [43]. This consequently caused a rise in available surface area for enhanced water absorption, better hydration and swelling, and high WHC in the case of WB-DF. SEM analysis also demonstrated rising tendency in surface tension strength as well as WB-DF porous matrix structure, which further enhanced WHC as compared to C-DF and blueberry residue.

The OHC values were measured for blueberry residue, C-DF, and WB-DF, and the results are presented in Figure 3B. The OHC values for all fractions were found in the range of 0.96–3.14 g/g. The lowest OHC value was exhibited by blueberry residue (0.96 g/g), whereas the WB-DF had the highest OHC value of 3.14 g/g. C-DF had an OHC of 1.97 g/g, which was significantly (*p* ≤ 0.05) higher when compared to that of blueberry residue. More oil molecules may absorb as a result of the cross-linked structures that emerge inside fiber chains following cross-linking treatment [16]. In addition, the OHC results were consistent with the morphological analyses, which suggested that the increased porosity of the DF structure, better particle dispersion, and a rougher surface led to enhanced oil absorption and retention by blueberry residue DF. The WHC and OHC are not only determined by DF thickness, viscosity, and total charge density, but are also dependent on the presence of hydrophobic and hydrophilic groups in the structure of DF particles. WB-DF had the highest OHC, owing to exposure and retention of hydrophobic groups, whereas the increased oil penetration in DF molecules was also facilitated by hydrophobic molecules, hence, leading to the prevention of oil loss. This property has peculiar significance with respect to food processing applications, whereby texture and shelf life of foodstuffs are improved.

The WSC values were determined for all samples and the results are graphically demonstrated in Figure 3C. The WSC values for all fractions ranged from 2.01 to 16.92 mL/g. The lowest WSC value was exhibited by BR (2.01 mL/g), whereas the WB-DF had the highest WSC value of 16.92 mL/g. C-DF had a WSC of 5.76 mL/g, which was significantly (*p* ≤ 0.05) higher when compared to that of blueberry residue. According to Hazarika and Sit [47], these occurrences could be caused by the formation of cross-linked structures inside fiber chains following C treatment, which could lead to more water molecules becoming entangled. An increase in WSC was also observed by Zheng et al. [48] in millet bran DF following C treatment. In addition, the enhanced WSC in the case of WB-DF was ascribed to the porous structure, wrinkled surface, hydrogen bonding disruption, and smaller particle size [15,29]. Among all samples, WB-DF had the highest WHC, OHC, and WSC, possibly owing to its more porous surface structure (Figure 1) and lower particle size (Table 3).

#### 3.9.2. BAC

**Figure 4:**
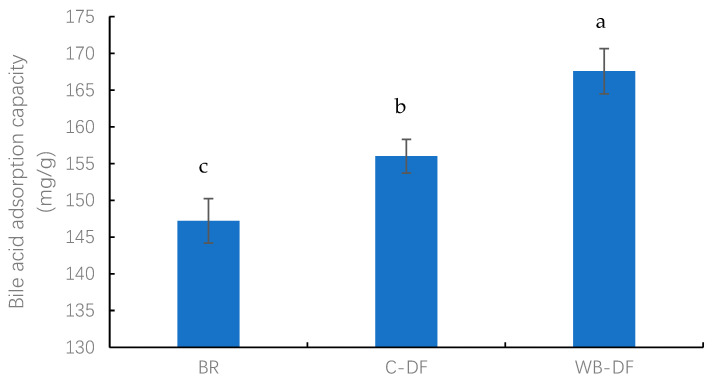
Bile acid adsorption capacity of BR, C-DF, and WB-DF. BR: blueberry residue; C-DF: cross-linked dietary fiber of blueberry residue; WB-DF: wet ball-milled dietary fiber of blueberry residue. Different letters above the bars indicate significant differences between the mean values (*p* ≤ 0.05).

The BAC values were determined for blueberry residue, C-DF, and WB-DF, and results are graphically demonstrated in Figure 4. The BAC values for all samples ranged from 147.21 to 167.58 mg/g. The lowest BAC value was exhibited by the blueberry residue (147.21 mg/g), whereas the highest BAC value of 167.58 mg/g was observed in WB-DF. C-DF had a BAC of 156.06 mg/g, which was significantly (*p* ≤ 0.05) lower compared to that of WB-DF (167.58 mg/g). The results showed that DF modification methods exhibited a significant effect on BAC values. It was also reported by previously published reports that the BAC of blueberry residue DF is affected by the DF’s gel characteristics and anionic groups’ contents. Moreover, IDF’s cholesterol-binding ability is also linked with internal IDF structure, charge densities, surface properties, particle size, and hydrophobic groups’ interactions [30,49].

#### 3.9.3. CAC

**Figure 5:**
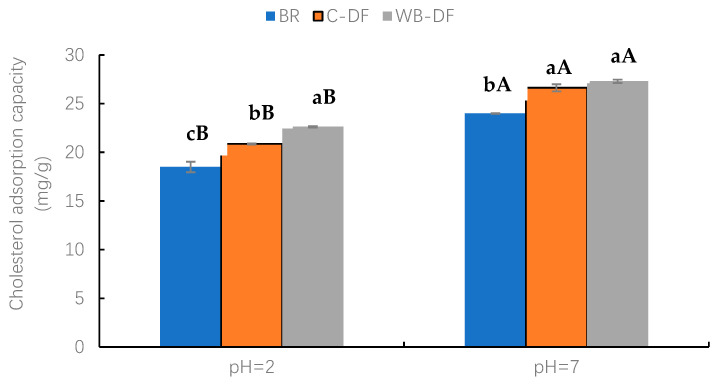
Cholesterol adsorption capacity of BR, C-DF, and WB-DF. BR: blueberry residue; C-DF: cross-linked dietary fiber of blueberry residue; WB-DF: wet ball-milled dietary fiber of blueberry residue. Means with different lowercase letters (a–c) above each group of bars (within the same group) indicate significant differences (*p* ≤ 0.05). Means with different uppercase letters (A–B) above each type of treatment (between groups) are significantly different (*p* ≤ 0.05).

The CAC values were determined for blueberry residue, C-DF, and WB-DF, and results are graphically demonstrated in Figure 5 at a pH of 2.0 and 7.0. Two factors, modification methods (BR, C-DF, and WB-DF) and pH (2, 7) and their interaction were evaluated. At pH 2.0, WB-DF had the highest CAC value of 22.62 mg/g, followed by C-DF (20.86 mg/g) and blueberry residue (18.51 mg/g). The highest CAC value in WB-DF could be ascribed to the structural framework loosening of blueberry residue DF when exposed to WB treatment. The CAC values were also assessed for all samples at pH 7.0. Both WB-DF and C-DF exhibited comparable CAC values of 27.31 mg/g and 26.63 mg/g, respectively, with no significant (*p* > 0.05) difference. The CAC value of blueberry residue was 24.01 mg/g at pH 7.0. All the modification methods had higher CAC values at pH 7 compared to those at pH 2. Nonetheless, the CAC results of WB-DF suggest that an increased number of hydrophilic groups interacted with water molecules after exposure to WB, leading to higher hydration (WSC and WHC) properties. The highest CAC values in WB-DF could be attributed to various influences, such as (1) enhanced network structure to facilitate additional absorption, (2) lower particle size, (3) breakdown of fibrous polymers after exposure to shearing force of milling, and (4) enhanced specific surface area. This caused a rising tendency in honeycomb structure formation, which promoted more cholesterol binding [30]. Therefore, WB treatment could be a proper modification method for enhanced CAC.

#### 3.9.4. GAC

**Figure 6:**
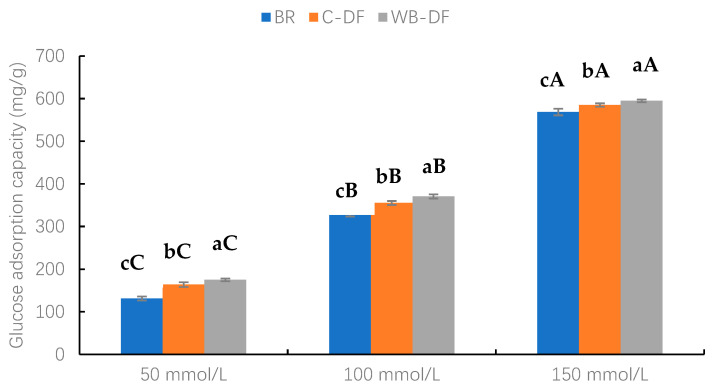
Glucose adsorption capacity of BR, C-DF, and WB-DF. BR: blueberry residue; C-DF: cross-linked dietary fiber of blueberry residue; WB-DF: wet ball-milled dietary fiber of blueberry residue. Means with different lowercase letters (a–c) above each group of bars (within the same group) indicate significant differences (*p* ≤ 0.05). Means with different uppercase letters (A–C) above each type of treatment (between groups) are significantly different (*p* ≤ 0.05).

The GAC values of blueberry residue, C-DF, and WB-DF samples were assessed at glucose concentration levels ranging from 50 to 150 mmol/L and results are demonstrated in Figure 6. Two factors, modification methods (BR, C-DF, and WB-DF) and glucose concentration (50, 100, and 150 mmol/L), along with their interaction, were evaluated. As observed, an increasing glucose concentration resulted in increased GAC values. Furthermore, it was evident from the results that, at all glucose concentration ranges (50–150 mmol/L), the highest GAC values were exhibited by WB-DF (174.82–594.93 mg/g), followed by C-DF (163.93–585.12 mg/g), and blueberry residue (131.14–568.78 mg/g), respectively. It had already been reported by Ma et al. [49] that DF’s particle size exerts significant influence on GAC values. The enhanced GAC values in WB-DF could be attributed to rising tendencies in cracks, porosity, blueberry residue DF surface area, and enhanced exposure of polar groups, which may promote glucose molecules entrapment with structural configuration of fiber [30]. Moreover, another probable reason for the highest GAC values might be that the highest WHC of WB-DF and WB exposure led to easier access of hydrophilic glucose molecules to the fiber network, promoting their binding [48]. Conclusively, the results of this study showed that GAC values of blueberry residue DF were significantly increased after modification through WB treatment.

## 4. Conclusions

This study represented the first demonstration of wet ball-milling treatment effectively resulting in enhanced structural, physicochemical, functionality, and rheological properties of blueberry residue DF compared to that of other cross-linked dietary fiber of blueberry residue and untreated blueberry residue. Generally, wet ball-milled dietary fiber of blueberry residue exhibited more intricate and looser microstructural attributes. More functional groups were exposed after exposure to wet ball-milling treatment which in turn led to significant improvement in hydration (water-holding capacity, oil-holding capacity, and water-swelling capacity), functionality (bile acid-adsorption capacity, cholesterol adsorption capacity, and glucose adsorption capacity), and rheological properties. It was suggested by the results of this study that wet ball-milled dietary fiber of blueberry residue exhibited remarkable potential to be developed as a food fortifier and natural anti-obesity health food. Conclusively, this study provided valuable insight regarding improvements in blueberry residue dietary fiber and, hence, provided a theoretical basis for developing functional foods fortified with blueberry residue dietary fiber.

## Figures and Tables

**Table 1:** Yield and proximate composition of BR, C-DF, and WB-DF.

	DF Yield (%)	DF Yield (%)	Ash (%)	Fat (%)
BR	-	0.94 ± 0.05a	2.70 ± 0.16a	0.26 ± 0.03a
C-DF	71.78 ± 1.61a	0.44 ± 0.04c	1.69 ± 0.05c	0.13 ± 0.01c
WB-DF	40.62 ± 1.24b	0.54 ± 0.04b	1.90 ± 0.09b	0.17 ± 0.01b

Means ± SD (n = 9) with different letters (a–c) within the same column indicating significant differences (*p* ≤ 0.05). BR: blueberry residue; C-DF: cross-linked dietary fiber of blueberry residue; WB-DF: wet ball-milled dietary fiber of blueberry residue.

**Table 2:** Apparent viscosity, consistency coefficient, and flow behavior index for BR, C-DF, and WB-DF.

	Apparent Viscosity 25 1/s [γ̇, mPa s]	Consistency Coefficient [K, mPa s]	Flow Behavior Index [n, -]
BR	19.87 ± 2.14c	183.10 ± 3.86c	0.32 ± 0.04a
C-DF	33.80 ± 4.18b	387.94 ± 5.95b	0.27 ± 0.02a
WB-DF	77.08 ± 3.97a	697.35 ± 6.94a	0.31 ± 0.03a

Means ± SD (n = 9) with different letters (a–c) within the same column indicating significant differences (*p* ≤ 0.05). BR: blueberry residue; C-DF: cross-linked dietary fiber of blueberry residue; WB-DF: wet ball-milled dietary fiber of blueberry residue.

**Table 3:** The particle size and ζ-potential for BR, C-DF, and WB-DF.

	Particle Size (nm)	ζ-Potential (mV)
BR	531.2 ± 23.5a	−20.1 ± 4.30a
C-DF	570 ± 24.2a	−28.7 ± 1.68b
WB-DF	451 ± 16.8b	−34.0 ± 4.13c

Means ± SD (n = 9) with different letters (a–c) within the same column indicating significant differences (*p* ≤ 0.05). BR: blueberry residue; C-DF: cross-linked dietary fiber of blueberry residue; WB-DF: wet ball-milled dietary fiber of blueberry residue.

**Table 4:** Molar ratio of monosaccharide components for BR, C-DF, and WB-DF.

	Man	Rha	GalA	Glc	Gal	Xyl
BR	1.00	3.00	5.80	40.00	2.43	1.23
C-DF	1.00	3.75	17.99	10.05	3.25	2.52
WB-DF	1.00	3.08	14.90	5.87	3.13	2.50

BR: blueberry residue; C-DF: cross-linked dietary fiber of blueberry residue; WB-DF: wet ball-milled dietary fiber of blueberry residue.

## Data Availability

The original contributions presented in this study are included in the article. Further inquiries can be directed to the corresponding author.
